# Three-Dimensional Anisotropic Metamaterials as Triaxial Optical Inclinometers

**DOI:** 10.1038/s41598-017-02865-z

**Published:** 2017-06-02

**Authors:** Kriti Agarwal, Chao Liu, Daeha Joung, Hyeong-Ryeol Park, Sang-Hyun Oh, Jeong-Hyun Cho

**Affiliations:** 1 Department of Electrical and Computer Engineering, University of Minnesota, Minneapolis, MN 55455 USA; 2 Department of Physics, Chung-Buk National University, Cheongju, 28644 South Korea

## Abstract

Split-ring resonators (SRRs) present an attractive avenue for the development of micro/nano scale inclinometers for applications like medical microbots, military hardware, and nanosatellite systems. However, the 180° isotropy of their two-dimensional structure presents a major hurdle. In this paper, we present the design of a three-dimensional (3D) anisotropic SRR functioning as a microscale inclinometer enabling it to remotely sense rotations from 0° to 360° along all three axes (X, Y, and Z), by employing the geometric property of a 3D structure. The completely polymeric composition of the cubic structure renders it transparent to the Terahertz (THz) light, providing a transmission response of the tilted SRRs patterned on its surface that is free of any distortion, coupling, and does not converge to a single point for two different angular positions. Fabrication, simulation, and measurement data have been presented to demonstrate the superior performance of the 3D micro devices.

## Introduction

Microscale inclinometers^1, 2^, (tilt sensors) capable of sensing angular motion along one or two orthogonal directions are currently used on a wide scale in industry^3–7^. In order to sense changes in the angle along three orthogonal axes, three separate inclinometers need to be mounted on a single substrate^8, 9^. However, this can result in mechanical interference, which is further amplified when they have similar resonant frequencies^10, 11^. Efforts have been made towards developing multi-axial sensors based on a liquid pendulum acting as a proof mass^12^. However, they suffer from lack of integration with conventional microscale fabrication techniques and require complex strategies for sealing electrolytes and electrode immersion in order to reduce the effects of internal and external factors on the performance of the inclinometer^13, 14^. The rapid saturation of the output voltage in these electrolytic inclinometers also imposes a limitation on the maximum angle resolved (60°)^15^. MEMS (microelectromechanical systems) capacitive inclinometers overcome the environmental factors affecting electrolytic sensors^16^, but suffer from severe performance degradation induced by defects produced during the fabrication process^17^, electromagnetic interference, and fringe effects resulting^12^ resulting in a limited range^18, 19^. Optical inclinometers^20–24^ have been studied as an alternative to the MEMS based devices, due to their ease of on-chip integration^25^. However, these optical sensors are capable of reliably detecting changes in angular motion and position only in one or two orthogonal directions^26^ for a maximum range of −45° to +45°^27, 28^. Recently, sensors capable of resolving rotations across all three-dimensions simultaneously have been studied for precise measurement of the position and orientation of medical microbots^29–32^. Microbots loaded with diverse sensors are envisioned to provide simultaneous *in-vivo* detection, diagnosis, and drug delivery thus allowing for fast, minimally invasive treatments^33, 34^. For such devices it is necessary to collect, transmit, and store a wide range of data as they traverse through the body. However, given the size of these microbots, the total amount of power and area available is extremely limited, which presents the need for small navigation systems (rotation sensors) that could be remotely monitored in order to maximize the power and area available to other circuit components. It is also essential to realize reliable sensors that are not affected by the temperature and pressure of the environment surrounding the microbots^35^. Thus, a need exists for research into the development of micro-machined angular sensors based on novel designs that meet the environmentally invariant, low power, and tri-axial remote sensing requirements.

**Figure 1:**
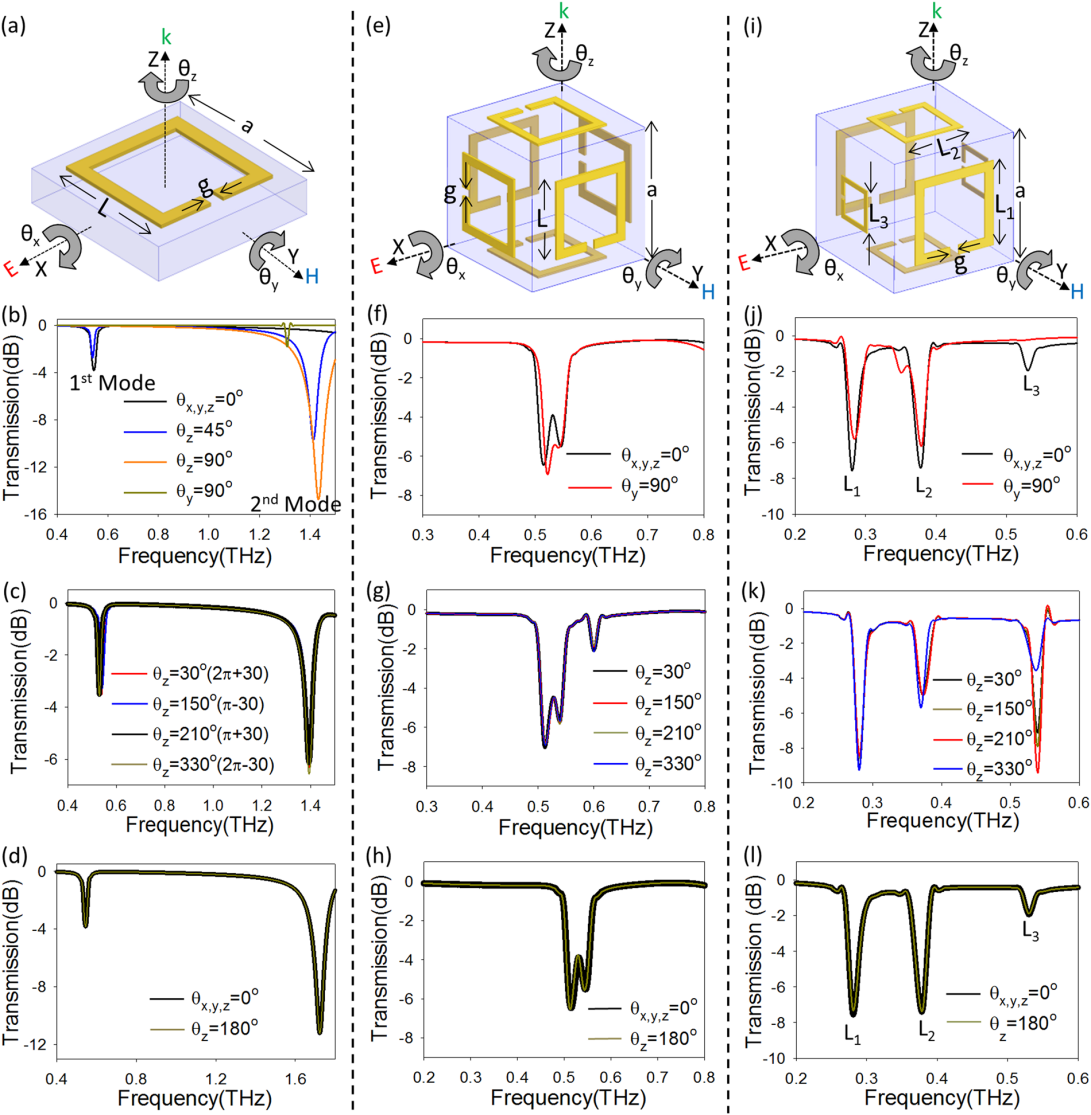
Illustration of two- and three-dimensional split-ring resonators (SRRs) and their simulated transmission response. (**a**) A conventional, two-dimensional SRR with L = 36 µm, g = 4 µm, and a = 48 µm, that can be rotated along the X-, Y-, and Z-axis at angles θ_x_, θ_y_, θ_z_ degrees, respectively. At the initial position the resonator has the wave at normal incidence, magnetic field (H) polarized perpendicular to the gap and electric field (E) polarized parallel to the gap. (**b**) Showing the weak transmission observed when rotated about Y-axis (θ_y_) as opposed to the strong first mode (θ = 0°) and second mode (θ_z_ = 90°) (**c**) Overlap of the resonance at 0.52 THz for rotations of 30°, 150°, 210°, and 330° about the Z-axis. (**d**) Transmission for the initial position (θ = 0°) and (θ_z_ = 180°), proving the isotropy of the transmission for any angle θ and nπ ± θ (n = 1, 2). (**e**) A cubic three-dimensional split ring resonator with L = 36 µm, g = 4 µm, and a = 110 µm, capable of maintaining a high SNR when rotated along all three-axes. (**f**–**h**) Simulated transmission response of the cube showing, (**f**) the high but ambiguous transmission response when rotated about Y-axis, (**g**) an isotropic transmission response for any angle θ and nπ ± θ (n = 1, 2) similar to the 2-D resonator, and (**h**) perfect overlap of the transmission at 0° and 180°. (**i**) A cubic rotation sensor with varying resonator length along each axis with L_1_ = 72 µm, L_2_ = 54 µm and L_3_ = 36 µm while ‘g’ and ‘a’ are kept constant as before. (**j**–**l**) Simulated transmission response of the cube showing, (**j**) the ability of the cube to maintain the high transmission for Y-axis rotation, (**k**) significant changes in transmission between rotations of angle θ, and nπ ± θ (n = 1, 2), and (**l**) special case of a 180° rotation that perfectly overlaps the θ = 0° initial position.

A microscale split-ring resonator (SRR, Fig. 1a) is a great candidate for the design and fabrication of optical inclinometers (angular sensors), since its resonant frequency and transmission amplitude are independent of environmental parameters such as temperature and pressure, and it also allows remote sensing without on-chip power consumption. The resonant frequency of a SRR is dependent on the shape of the resonator, length of the split, and permittivity of the material within the split^36^; whereas, the strength of resonance, i.e. the transmission amplitude at the resonant frequency, depends on the angle of incidence of the incoming light, *k*, and the dielectric constant of the surroundings^37^. The strong dependence of the SRR resonance on the aforementioned has been utilized for the development of a wide range of sensors to detect micro-organisms^38–40^, strain^41, 42^, dielectric constants^43^, and displacement^44^. A major limiting factor in the further implementation and commercialization of these devices have been disadvantages arising from their low quality factor leading to a low sensitivity. However, research into design of the conventional C-shaped SRR using nanopillar^45^, asymmetry of the split^46^, and modification of coupling within SRR arrays^47^ have led to an increase in the quality factor by a factor of 30 and a corresponding 10 fold increase in sensitivity.

The transmission spectrum of a SRR defined on a planar substrate (Fig. 1a) shows that as the angle of rotation (θ_z_) increases, the first mode amplitude reduces, whereas the second mode amplitude increases until at 90° the resonator is completely in the second mode (Fig. 1b). As the angle of incidence is increased beyond 90° to 180°, the reverse phenomenon takes place, where the first mode amplitude increases and the second mode amplitude decreases, making the conventional SRR 180° isotropic^48^. Thus, limiting the application of these sensors due to their inability to distinguish between positive and negative angles and a range limited from 0° to 90°, such that rotations of 30°, 150°, 210°, and 330° (nπ ± θ, n = 1, 2) all transduce the same change in transmission (Fig. 1c). A recent design introduced a tapered U-shaped SRR consisting of two resonators where the area of overlap between the two changed in response to a rotation, resulting in a change in coupling between the two resonators, thereby also changing the resonant frequency^49^. The tapered shape allowed distinguishing between positive and negative angles and increased the sensing range from 90° to 180°. However, the two tapered SRRs could only perform measurements along one axis and were unable to overcome the limitation of 180° being the maximum angle that could be resolved. Angular sensors based on SRRs defined on a planar substrate (Fig. 1a) are thus plagued by an ambiguity in the angle sensed such that a rotation of angle θ and that of 180 + θ produces the same change in resonance of the structure (Fig. 1d). For three-axis sensing, the uniaxial nature of these planar sensors also requires the fabrication of three independent sensors directed along the X-, Y-, and Z-axes respectively, each with their own light source, thereby decreasing the packaging density of these devices for applications like microbot positioning systems, where a three-dimensional (3D) rotation measurement is essential for calculating the exact position and spin of a microbot during *in-vivo* experiments.

In this paper, we present a proof-of-concept of an optical inclinometer with 3D anisotropic SRR structures defined on a polymeric cube that overcomes the limited range of the two-dimensional (2D) SRR structures defined on a planar substrate. The anisotropic 3D design enables the SRR to remotely sense rotations from 0° to 360° along all three axes through the measurement of the transmission variation at the resonant frequencies under rotation, by employing the geometric property of a 3D structure. Simulation and measurement data have been provided to demonstrate the superiority of these 3D resonant structures over conventional inclinometers in measuring the angle of rotation in a 3D space. Thus, inclinometers based on these 3D structures have the potential to exceed the current limitations of the MEMS and optical angular motion sensors arising from vibrating proof masses^10, 11^ and limited sensing range^27, 28^.

## Results

### Analysis

When an SRR defined on a planar substrate (Fig. 1a) is rotated along the X- and Y- axes, the resonance strength reduces such that when the incident wave is parallel to the resonator (θ_y_ = 90°), a very weak resonance is observed (Fig. 1b). In order to measure rotation angles of the structure about the X-, Y-, and Z-axes without a compromise in signal to noise ratio (SNR), the cubic point of symmetry can be leveraged^50–52^ by positioning identical 2D planar resonators on a dielectric cube^53^ (Fig. 1e). By patterning SRRs on cubic (3D) structures to form a face-centered cubic lattice, rotations about any axis can be performed while maintaining at least two resonators that have the incident wave perpendicular to them, giving a measurable transmission for θ_y_ = 90° (Fig. 1f), unlike the case (Fig. 1b) of SRRs defined on a planar substrate (Fig. 1a). However, as seen by the transmission spectrum when resonators of fixed length, *L*, are patterned on these cubic structures (Fig. 1e), the transmission response remains invariant for 180° of rotation around any axes, similar to the 2D structure shown in Fig. 1a, (θ° is not distinguishable from θ ± 180° and −θ°), such that the transmission spectrum for 30°, 150°, 210°, and 330° perfectly overlap each other (Fig. 1g). Thus, even for the case of the 3D cube with SRR patterned on its surface (Fig. 1e), the sensing range of the cube is limited to 180° (Fig. 1h).

To overcome the limited sensing range of the resonators defined on the cubic structure, the cube can be fabricated with resonators of varying sizes. By arranging resonators of length L_1_, L_2_, and L_3_ on each face (Fig. 1i), we can obtain a cubic structure with resonators of three different resonant frequencies (Fig. 1j); the resonators on the opposite faces of the cube are kept at the same length. As shown in Supplementary Fig. S1, the resonator with L_1_ = 72 µm resonates at a 1^st^ mode resonant frequency of f_r_L1_ = 0.28 THz, whereas the resonators with L_2_ = 54 µm and L_3_ = 36 µm have 1^st^ mode frequencies as f_r_L2_ = 0.38 THz and f_r_L3_ = 0.52 THz, respectively. This leads to a transmission response (Fig. 1j) where the first three peaks at different frequencies represent the fundamental (1^st^ mode) resonance of each resonator; by evaluating the amplitude of each of the three peaks, the angle of rotation could be found. Unlike the uniform SRRs defined on the cube shown in Fig. 1e, for a cube with SRRs of varying lengths, rotation of any angle θ° is distinguishable from θ ± 180° and -θ° such that the transmission spectra for 30°, 150°, 210°, and 330° are distinguishable from each other (Fig. 1k). However, even with the ability to distinguish rotations along each axis, the range of the cubic structure (Fig. 1i) remains limited to 180° as the resonator gives the same response for rotations of angle 0° and 180° along any axis (Fig. 1l). Hence, the cubic inclinometer with resonators of varying length is able to sense rotations from 0–360° about all three-axes except for the special case where, θ_x_ = θ_y_ = θ_z_ = 180°.

**Figure 2:**
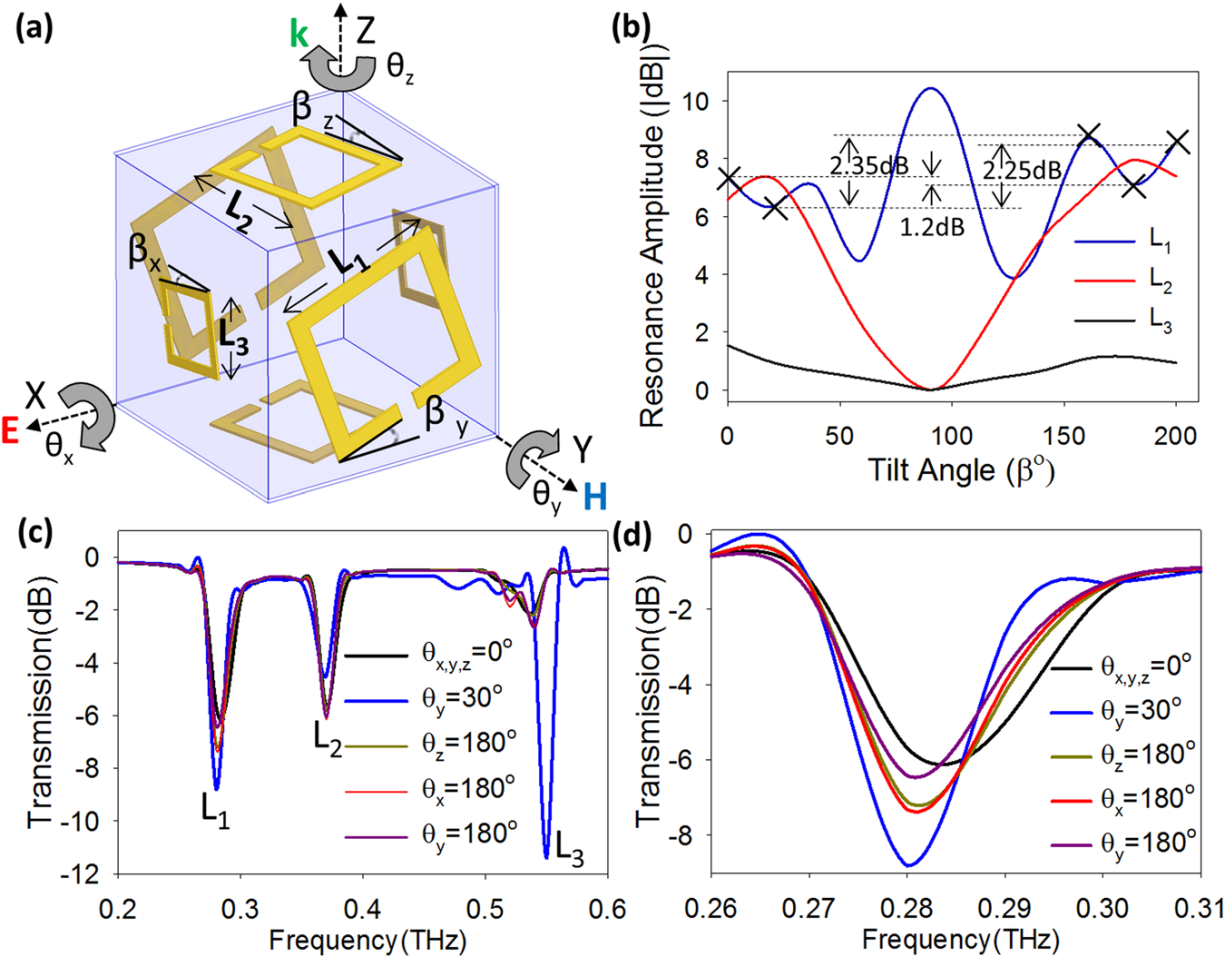
Effect of the angular offset on the anisotropy of the 3D inclinometer. (**a**) Cubic rotation sensor with an angular offset of β_x_, β_y_, and β_z_ for each resonator L_3_, L_1_, and L_2_, respectively. (**b**–**d**) Simulation results for the cubic sensor. (**b**) The variation in the first mode resonance strength as a function of the angular offset obtained by placing only 1 of the 3 resonators at a single time to avoid couplings; L_2_ and L_3_ have a 180° period but the L_1_ resonator has no symmetry in its transmission. (**c**) Graph showing the anisotropic effect of adding angular offset; the resonator L_1_ demonstrates a significant change in amplitude for a 180° rotation about any axis. (**d**) Zoomed in graph of (**c**) showing the variance in transmission of the L_1_ resonator.

In order to solve the above limitation, it is necessary to break the 180° symmetry in the transmission response for at least one of the resonators, such that the entire cube loses its 180° isotropy. If the resonators of length L_1_, L_2_, and L_3_ are tilted at angles β_*y*_, β_*z*_, β_*x*_ about their axis, respectively (Fig. 2a), they undergo a change in their transmission amplitude. The change in resonant behaviors of the three resonators obtained using the ANSYS high frequency structural simulator, HFSS (version 13.0.1), is given in Fig. 2b, where it can be clearly seen that two resonators (L_2_ and L_3_) demonstrate a period of 180°, whereas the L_1_ resonator does not demonstrate any symmetry in its transmission amplitude. When the resonators with L_2_ = 54 µm and L_3_ = 36 µm rotate along the Z- and X-axis (when β_*z*_ and β_*x*_ increase), respectively, they undergo a change in the transmission; the first mode amplitude decreases as the resonators are tilted to an angle of 90° and increases beyond that (Fig. 2b). The resonator L_2_ and L_3_ transition from their initial configuration of first mode to second mode when tilted from 0° to 90° and vice-versa from 90° to 180°. A lower transmission is obtained for the L_3_ resonator since it has the incident wave (k) parallel to the plane of the resonator. The resonator with L_1_ = 72 µm defined along the Y-axis is also parallel to the incident wave, but the resonator (L_1_) undergoes a resonance condition with the plane of the magnetic field (H) lying parallel to the plane of the resonator. This polarization produces strong electrical and magnetic resonances within the SRR at its fundamental resonant frequencies^54, 55^. However, the resonance does not follow the conventional 180° period as it did for L_2_ and L_3_ (Fig. 2b). The electrical resonance disappears only for β_*z*_ = 0°, whereas the magnetic resonance never reaches 0 decibels (dB). For all increments of β_*y*_ from 0° to 200°, a resonance amplitude ranging from 10 to 4 dB was seen for the 1^st^ mode of the L_1_ resonator (Fig. 2b). The electrical resonance (second mode) of the L_1_ resonator occurs at 0.52 THz, same as the magnetic (first mode) resonance for the L_3_ = 36 μm resonator. The transmission amplitude at 0.52 THz is thus a superimposition of these two individual waveforms, the resonators cannot be called as coupled since they continue to resonate at their individual resonant frequencies as in the absence of the other. This superimposition of the waveforms further enhances the ability of the cubic structure shown in Fig. 1c, to distinguish angles for all values of θ and (nπ ± θ, n = 1,2) except for θ_x,y,z_ = 0° (0° and 180°) (Fig. 1l), where the electrical resonance (2^nd^ mode) for the L_1_ resonator goes to zero. Only at β_*y*_ = 0°, 180°, the transmission amplitude of the resonator (L_1_) with the magnetic field plane lying parallel to the plane of the resonator demonstrates a change of 1.2 (dB) (Fig. 2b), a small change that gets easily masked by the smallest coupling with the L_2_ and L_3_ resonators, forcing the perfect overlap of the transmission response as seen in Fig. 1l for θ_x,y,z_ = 0°, and θ_z_ = 180°. However, if the resonator is now tilted by an angle of β_*y*_ then, on an 180° rotation of the cube around the Y-axis from the initial position, the total rotation experienced by the L_1_ resonator changes. If β_*y*_ = 20° at the initial position (Supplementary Fig. S2a), then on rotating the cube around Y-axis by 180° (θ_y_ = 180°), the effective angle (β_*y*_´) of the L_1_ resonator changes to β_*y*_ *´* = 200° configuration (Supplementary Fig. S2b). As shown in Fig. 2b, the change in transmission between 20° and 200° is 2.25 dB, double the transmission change before. Similarly, when the cube is rotated by 180° along the X-axis (θ_x_ = 180°), the L_1_ resonator with β_*y*_ = 20° initially, now experiences a β_*y*_ = 160° configuration causing a total change of 140° in its position (Supplementary Fig. S2c) that corresponds to a change of 2.35 dB (Fig. 2b) in the transmission amplitude between the two configurations. Similarily, an 180° rotation about the Z-axis (θ_z_ = 180°) causes a 320° change in the position for the L_1_ resonator (Supplementary Fig. S2d) that causes a 5.45 dB change (Supplementary Fig. S3a) in the transmission amplitude. It could be argued that for a rotation of θ_y_ = −20°, and θ_y_ = 160°, the cubic structure will once again reach the non-tilted structure resulting in the original β_y_ = 0° and 180° configurations, respectively. However, when tilted about the Y-axis at β_y_ = 20°, the coupling of the L_1_ resonator with L_2_ and L_3_ changes, such that the small difference previously seen in the transmission response of the L_1_ resonator between β_y_ = 0° and 180° positions (1.2 dB in Fig. 2b) is no longer masked by the coupling with L_2_ and L_3_ resonators (Supplementary Fig. S3b), leading to a distinguishable amplitude difference of 2.66 dB between θ_y_ = −20°, and θ_y_ = 160°, overcoming any ambiguity in the transmission response. Each resonator is tilted about its axis to respond in a similar manner to all the polarization directions of the light, not only the one simulated here. The tilt angles (β_*x*_, β_*y*_, β_*z*_) are chosen such that maximum transmission is obtained at the initial cube position of θ_x,y,z_ = 0°.

Based on the theory addressed above, the 180° isotropic limitation of the SRR can be overcome and the cubic sensor is capable of resolving rotations of any angle θ about various axes and even for angles 180 ± θ° by introducing the angular offset (Fig. 2c and d). As clearly seen in the Fig. 2c, when the resonator is rotated from the initial position (θ_x,y,z_ = 0°) along the Z-axis (θ_z_) by an angle of θ_z_ = 180°, the L_1_ resonator shows a large change in the transmission amplitude, whereas the L_2_ and L_3_ resonators retain their initial transmission. On the other hand, when the resonator is rotated along the Y-axis (θ_y_) from its initial position by a random angle, the resonators L_2_ and L_3_ demonstrate a large change in the transmission amplitude (Fig. 2c). A lower change is seen (~2 dB) for the transmission amplitude of the L_1_ resonator (Fig. 2d) on 180° rotations than previously discussed (2.25 to 5 dB shown in Fig. 2b and Supplementary Fig. S3a, results obtained by placing only 1 of 3 resonators) due to the minor coupling between the planar resonators on each cubic face; any coupling of L_2_ and L_3_ with L_1_ resonator attempts to mask its change in transmission. To ensure that the resonant frequencies of any two resonators never interfere with each other, the SRRs (L_1_, L_2_, and L_3_) were simulated individually on the faces of the cubes for tilt angles from 0° to 360° in steps of 10°. As shown in Fig. 1, the first mode switches over to second mode when increasing the tilt angle from 0° to 90° and vice versa beyond 90°, thus, it is primarily the overlap of 1^st^ and 2^nd^ modes of various resonators that needs to be considered. E.g. the 2^nd^ mode of 72 µm L_1_ SRR should not overlap with 1^st^ mode of 36 µm L_3_ SRR etc. This implies that the 2^nd^ and 3^rd^ mode peaks seen in Supplementary Fig. S1 should not overlap with any of the first modes.

**Figure 3:**
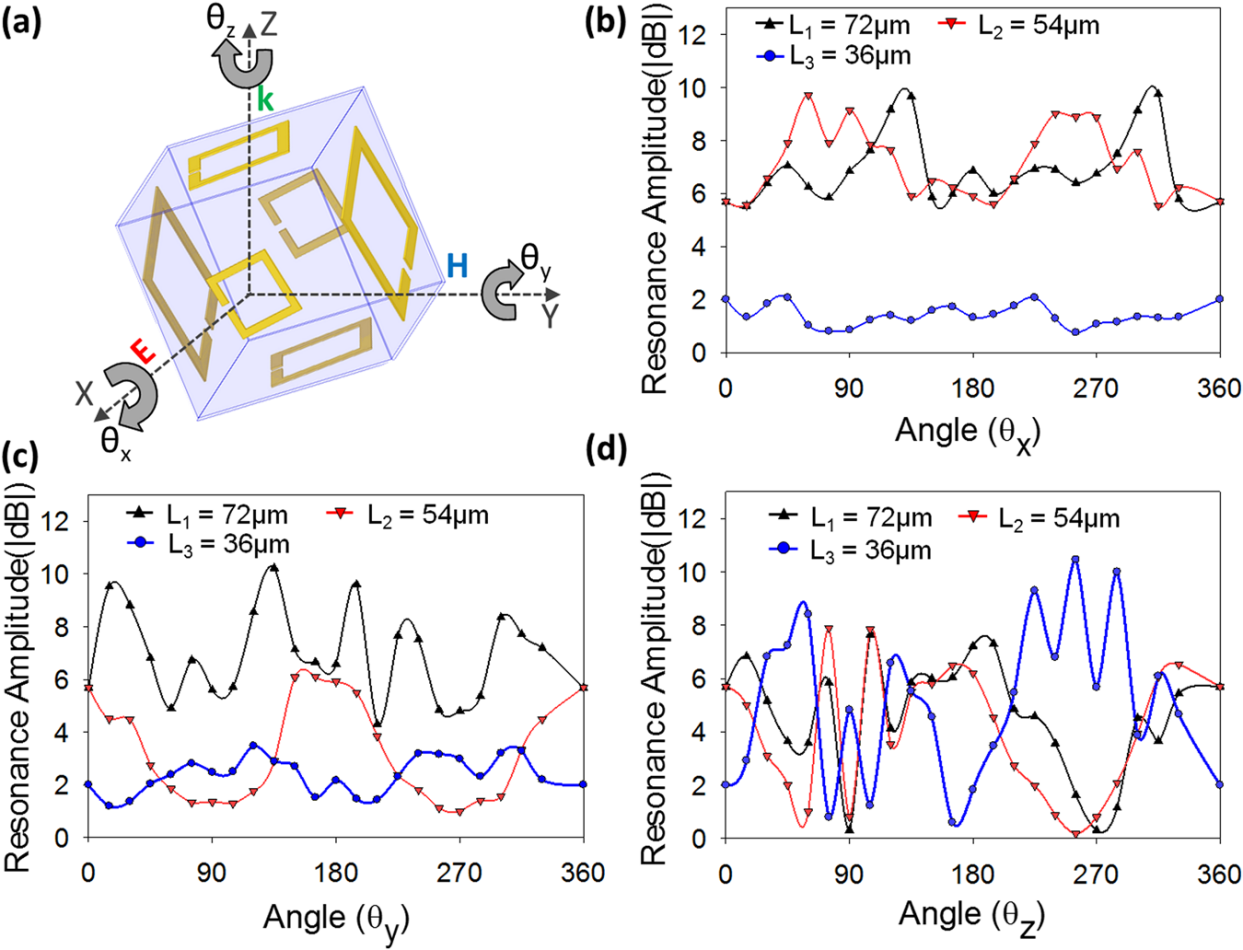
Simulation results for the rotation of the 3D cube. (**a**) Cubic inclinometer rotated about X-, Y-, and Z-axis at angles θ_x_, θ_y_, and θ_z_, respectively. Variation in resonance strength of each resonator obtained by simulating rotations along (**b**) X-axis, (**c**) Y-axis, (**d**) Z-axis in steps of 15° from 0–360° proving that for no two angles do the values of all three resonators overlap each other. The resonance amplitudes are obtained at the resonant frequencies of the L_1_ (0.28 THz), L_2_ (0.38 THz), and L_3_ (0.55 THz) resonators.

The cubic sensor is now simulated with rotations along each axis to form angles θ_x_, θ_y_, and θ_z_ along the X-, Y-, and Z- axes respectively as seen in Fig. 3a. The rotations along X- (Fig. 3b), Y- (Fig. 3c), and Z-axis (Fig. 3d) are performed from an angle of 0° to 360° in steps of 15° and the magnitude of the transmission peaks of each of the resonator at 0.28, 0.32, and 0.52 THz is measured. As can be seen from Fig. 3b–d, the transmission amplitude of each of the resonator varies when the cubic structure is rotated about each axis, by finding out the transmission amplitudes of all the resonators, the angle can be resolved. From the graphs in Fig. 3b–d, it can also be seen that the simulated transmission amplitude of all three resonators does not show the exact same value for any two rotation angles about X-, Y- or Z-axis, thus removing any ambiguity in the measurement of the angle of rotation of the cubic sensor.

### Fabrication

**Figure 4:**
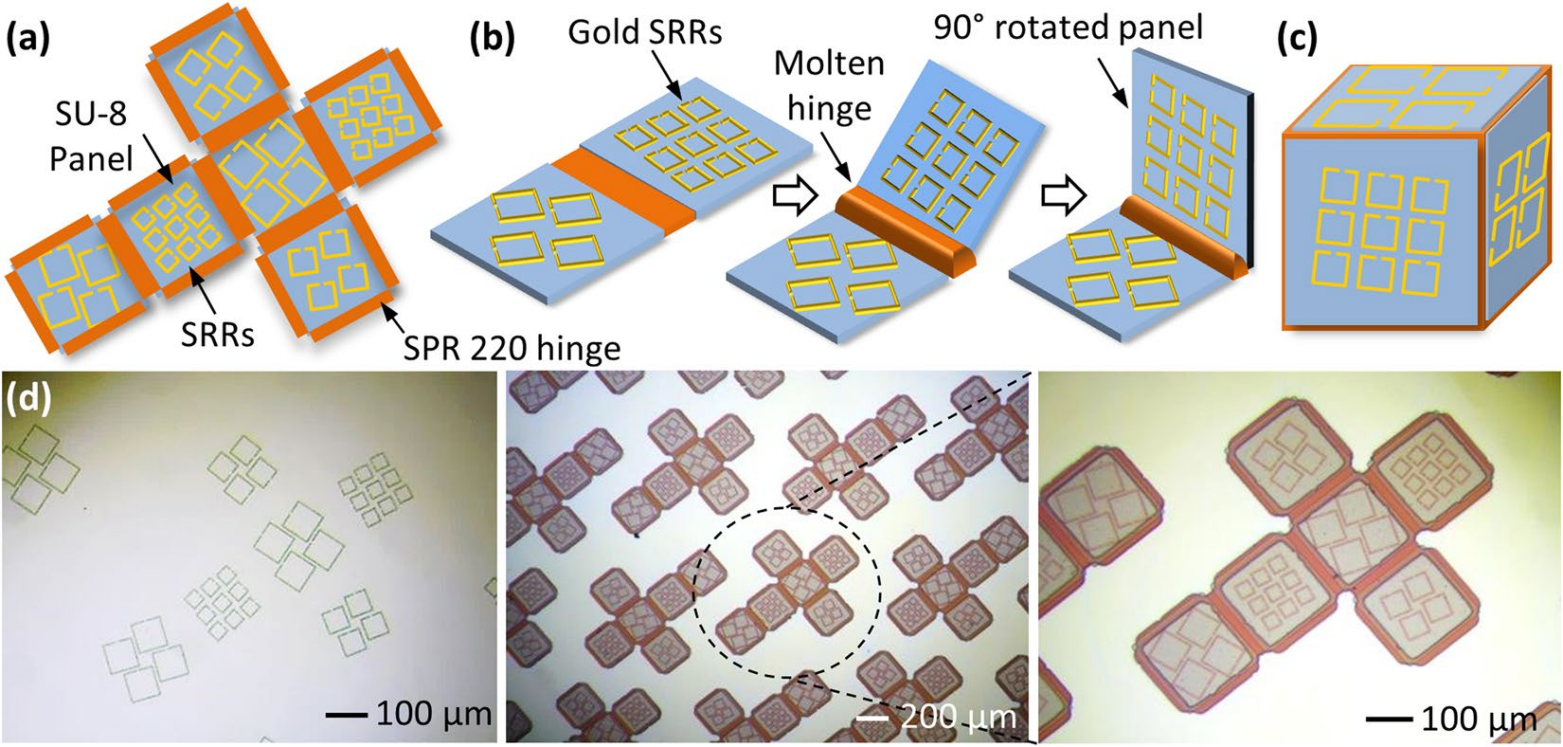
Fabrication process of the Triaxial inclinometer. (**a**) Illustration of the two dimensional planar configuration before the self-folding is initiated, showing the SU-8 panels, the Au SRRs and the SPR 220 hinges (**b**) Illustration of the self-folding process, demonstrating the melting of the hinge causing it to flow and generating a surface tension force that slowly lifts up and folds the panel to a 90° angle (**c**) Illustration of the folded 3D cubic structure through surface tension driven self-assembly, where the SU-8 panels form the faces of the cube once the hinges re-solidify (**d**) Optical images of the fabrication process for the cubic SRR inclinometer, starting with the patterning of Au SRRs, followed by the deposition of SU-8 panels and the SPR 220 hinges.

To experimentally demonstrate that an in-plane tilt within the resonators allows measurement of rotation angles from 0°–360° about all three-axes, a 500 μm sized cube with tilted SRRs was fabricated using a self-assembly process^56^. One of the important design considerations for the cubic sensor, is that the resonant frequencies of the 3 resonators (L_1_, L_2_, and L_3_) should not overlap or experience interference from the bandwidth of the resonance peaks of other resonators. Additionally, another important design consideration is that the resonant frequency should lie within the measurement range of the THz measurement system, and any additional components apart from the SRR (e.g. the cube) should be transparent within that frequency range. Several simulations were carried out for varied lengths of the resonator to find the optimum value of the resonant frequencies of the L_1_, L_2_, and L_3_ resonators and the corresponding lengths. For the cubic sensor, the SRR lengths were chosen as 72 µm, 54 µm, and 36 µm since their 1^st^ mode resonance does not spectrally overlap (Supplementary Fig. S1). Due to the decay in the transmission of these resonators when tilted about their axis, the tilt was chosen so as to obtain a high transmission at the initial position of 0°. Moreover, distinct values need to be given for the three tilt angles to break the 180° coupling symmetry. The resonators measuring 72 µm, 54 µm, and 36 µm patterned on a cubic surface with SU-8 2010 (MicroChem) panels and SPR 220–7.0 (MEGAPOSIT) hinges are tilted at angles βy = 20°, βz = 15°, and βx = 10° about their axis, respectively. The lowest tilt angle (βx = 10°) was assigned to the L_3_ SRR since at the initial position of the cube it has the lowest transmission amplitude of the 3 SRRs and decreases further with increasing tilt angle (Fig. 2b). For the L_2_ SRR, the transmission amplitude increases between 0°–20°. However, if we choose 20° as the tilt angle for the L_2_ SRR, then the L_1_ SRR would need to be 25–30° which will reduce its transmission amplitude significantly and due to its large dimension will make it difficult to accommodate on the face of the cube with a periodicity similar to the L_2_ and L_3_ SRR. Thus, the tilt angle of 15° was chosen for L_2_ SRR and a tilt angle of 20° for L_1_ SRR. 300 nm thick gold (Au) SRRs were deposited using an Au electroplating process. On top of the Au SRRs, 10 μm thick SU-8 panels were patterned and 21 μm thick hinges for the cube of SPR 220–7.0 positive photoresist are patterned between and around the panels. The 2D structure was thus defined with six SU-8 panels, Au SRR arrays on each face of the panel, and SPR 220 polymer hinges (Fig. 4a). For the self-assembly (folding) process, heat energy was applied to the 2D structures causing the SPR 220 hinges to reflow under high temperature and generate a surface tension force to fold the structure (Fig. 4b)^57^. On cooling, the SPR 220 hinges became solid again and secured the 3D cubic structure (Fig. 4c). As shown in Fig. 4d, by the optical image before the self-folding process, only the Au SRR is present as the resonant material. It should be noted that the entirely polymeric composition of the cube with SU-8 panels and SPR 220 hinges ensures that no noise or coupling of the SRRs to the cube frame exists, that can distort the transmission spectrum.

**Figure 5:**
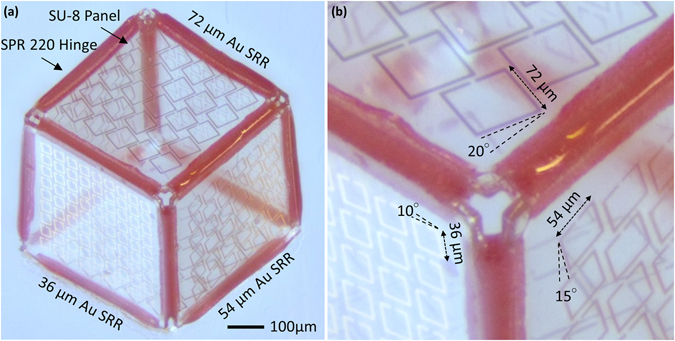
Optical Image of the 500 μm three-dimensional cubic inclinometer. (**a**) Three-dimensional cubic inclinometer with SU-8 panels, SPR 220 hinges, and Au SRRs of varying lengths with a tilt patterned on the surface. The cube consists of 5 × 5 array of 72 μm resonators (tilted at 20°), 6 × 6 array of 54 μm resonators (tilted at 15°), and 9 × 9 array of 36 μm resonators (tilted at 10°). Resonators on the opposite faces are identical. (**b**) A zoomed-in optical image of the 3D cubic inclinometer.

Following the reflow of the SPR 220 polymer hinge, the 2D net (Fig. 4d) forms the 3D cubic structure (Fig. 5), with two faces containing a 5 × 5 array of 72 µm (tilted at 20°), two faces containing a 6 × 6 array of 54 µm (tilted at 15°), and two faces containing a 9 × 9 array of 54 µm (tilted at 10°) Au SRRs present on the outside of the cube on top of the SU-8 panels. Through the application of uniform heat to the structures, the 2D planar structures can be folded uniformly by a surface tension force generated by the hinge material and transformed into 3D cubic structures within several seconds to a few minutes depending on the temperature applied to the hinges. Once the polymer hinges re-solidify, the resulting cubic structure is mechanically and thermally stable under 100 °C (the melting point of the re-solidified polymer hinges). Further details of the fabrication procedure can be found in Methods Section and Supplementary Information. The self-assembly of the structure can be visualized by the video file available in the Supplementary Information, where the folding of the 2D SU-8 and SPR220 structure is recorded after partially releasing the structure in air. The cubes can be folded in either air or water. SU-8 used as the panel material is a bio-compatible polymer^58–60^. On the other hand the hinge material (SPR 220) can cause *in-vivo* inflammation. However, the SPR 220 biocompatibility can be improved by surface modification techniques like anticoagulant immobilization on carbodiimide activated surfaces^61^ or deposition of a thin layer of Polyethylene (PE)^62^ on the hinges, the nanometer thickness of the biocompatible polymer layers does not impact the self-folding yield. The cubic structures can be further encapsulated within a biocompatible polymer shell along with other microbot components to avoid triggering an immune response.

### Simulation and measurement

**Figure 6:**
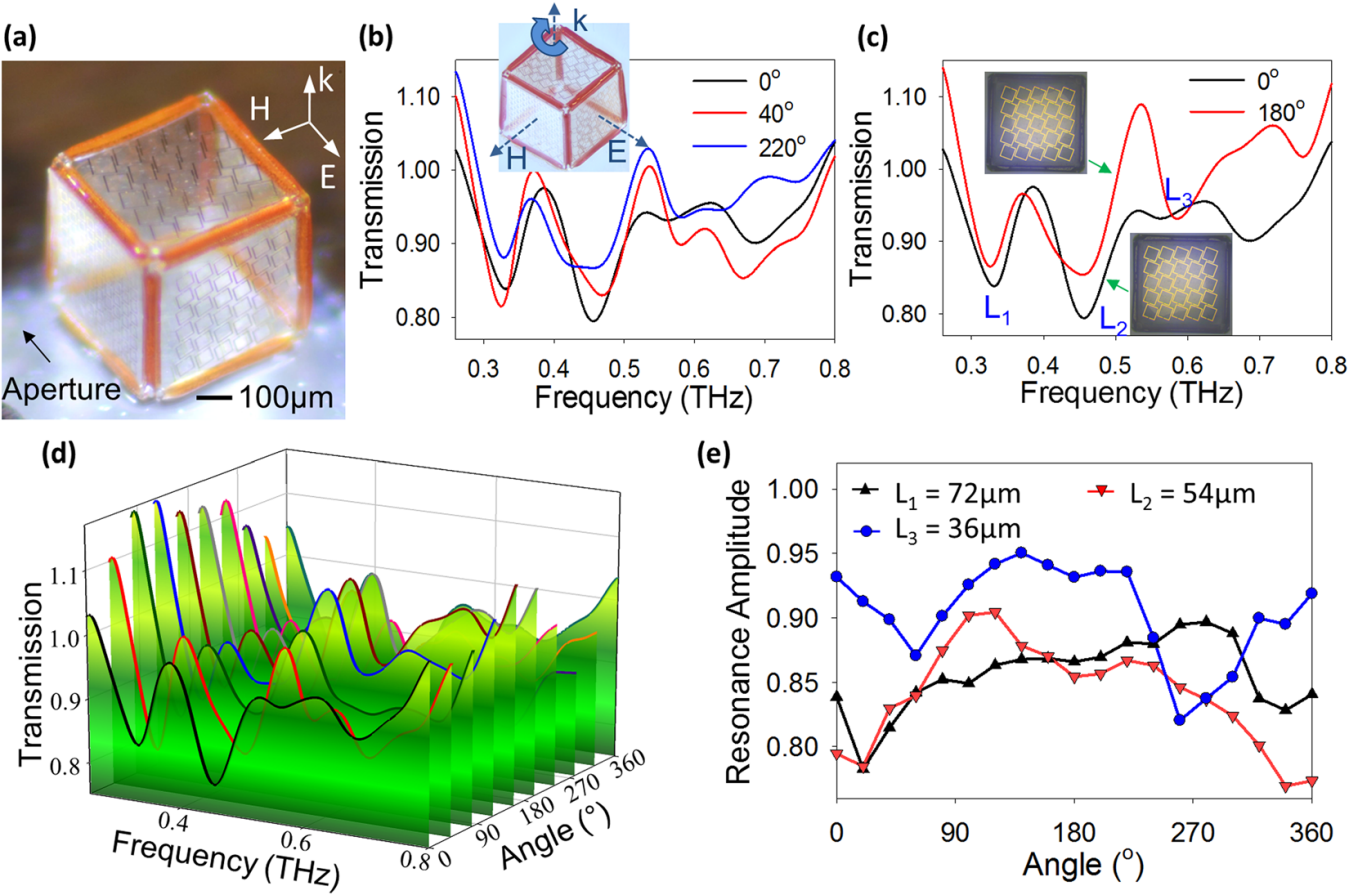
THz TDS measurement results for the cubic inclinometer. (**a**) Optical image of the experimental setup for THz transmission measurement, showing a 500 µm cube placed on the circular aperture with the inset showing the polarization direction of the THz wave. (**b**) Measured transmission response displaying the ability of the cube to distinguish various angles when rotated about any axis through a change in transmission. (**c**) Measured transmission response demonstrating a clear change in transmission and breaking of the 180° isotropy of the cube. (**d**) Measured frequency spectra of the cubic structure observed while rotating the cube about the direction of wave vector (k) in steps of 40° (**e**) Measured variation in the transmission response at the fundamental resonant frequency of the 3 resonators defined on the faces of a cube when rotated along the wave vector (k).

The 500 µm entirely polymeric cube with the Au SRR patterned on SU-8 panels and SPR 220 hinges was simulated using ANSYS Electromagnetics Suite 16.0.0 (formerly HFSS), keeping the light incident at 90° on the 54 µm resonator, magnetic field (H) polarized perpendicular to the 72 µm resonator, and electric field (E) polarized perpendicular to the 36 µm resonator (Supplementary Fig. S4a). Simulation of the transmission spectrum (S_21_ parameter) of the cube displayed three clear peaks at 0.33, 0.49, and 0.63 THz that correspond to the resonant frequencies of the 72 µm, 54 µm, and 36 µm resonators, respectively (Supplementary Fig. S4b). The fabricated sensor was characterized using terahertz time-domain spectroscopy (TDS) (0.2 THz to 0.8 THz). For the measurement, a single cube was attached to a piece of double-sided Scotch Tape (material transparent to THz wave) and was placed at the center of a circular aluminum aperture of diameter 3.8 mm (Fig. 6a). The presence of only the Au SRRs as the resonant material, as well as the entirely polymeric composition of the cube, also ensures that no interference or coupling exists that can distort the transmission spectrum^33^. A THz pulse generated from a commercial GaAs emitter passed through the aperture and cube and was received by a detector. The cube was placed such that at the initial position, the face of the cube with resonators of length 72 µm and resonant frequency 0.33 THz faces the incoming light. Further details of the simulation settings and measurement setup can be found in the Methods. The aperture of diameter 3.8 mm generates a cutoff frequency of 0.07 THz which results in maximum transmission drops of 0.8 at the initial 0° position (Fig. 6b). For the simulated cube, the transmission dropped to 0 at the resonant frequency for a 300 nm thickness Au SRRs (Supplementary Fig. S4), since the length of the vacuum box was six times smaller (0.6 mm). However, the measured transmission response (Fig. 6b) was found to have the same 3 resonant frequencies representing each resonator on the cubic surface and was in perfect agreement with the simulated spectrum (Supplementary Fig. S4b) since it is independent of the aperture size. To characterize the transmission changes based on different rotations, the cube was attached to a rotational mount so that the rotation of the cube along Z-axis could be precisely controlled.

Rotations performed for randomly chosen angles while keeping the 72 µm resonator facing towards the incoming wave, show the ability of the cube to distinguish between various angles. On rotating the cube by an angle of 180°, showed the reappearance of the three resonant peaks in the measurement spectrum, however, the transmission amplitude at the resonant frequency changes (Fig. 6c). On performing multiple rotations for varied angles along the Z-axis, the cube demonstrated a large change in transmission amplitude for at least one or more resonators (Fig. 6d). The cube was rotated in steps of 20° from 0° to 360° and the transmission amplitude of each of the resonators at their resonant frequency was plotted with respect to the angle of rotation. As seen in Fig. 6e, the amplitude of each resonator varies under rotation; at no point was the amplitude of all three resonators found to repeat itself for two different rotations. Thus, by finding the amplitude of all three peaks, the angle of rotation can be detected.

## Discussion

The measurement results prove that adding a tilt to the SRRs of varying lengths patterned on a cube provides us with a 3D anisotropic inclinometer that is capable of sensing angular changes from 0° to 360° without any ambiguity in the signal transduced. Measurement data for rotations along only the Z-axis is provided due to lack of a rotational mount capable of rotating also all three orthogonal axes. The THz TDS setup ensured that the resulting SNR was high enough (10^4^:1) such that the transmission could be measured with minimal error (10^−4^ for a transmission of 0.5). Taking into account the time taken to re-align and rotate the components manually (~4 min) between successive measurements, the measurement of the angular velocity sensing capabilities of these cubic structures presents a major hurdle due to lack of a mechanical clocking speed. However, given the repetition rate of 80 MHz (12.5 ns), with a 90 fs pulse width (Refer to Methods section for detailed setup) and the 20° angle increments, 3D SRR based angular velocity sensors could perform measurements with a resolution of 1.6 °/ns as opposed to current MEMS based sensors that are limited to 2000 °/s^63^.

To improve the sensitivity of the 3D inclinometer it is necessary to have a high quality (Q) factor. However, thin film-based traditional SRRs normally exhibit a low Q factor, which leads to a poor sensitivity. The sensitivity of the 3D inclinometer configured with SRRs might be enhanced by utilizing displacement current with the use of nanopillars, which boosts the quality factor up to about 400^45^. On the other hand, one of the drawbacks of the nanopillars-based SRRs is a weak resonance behavior leading to lower transmission amplitude (|S_21_|). Without sacrificing the resonance behavior, a high Q factor may be achieved at high resonance modes by employing Fano and quadrupole resonances^64–66^. However, since these resonances are different in behavior (confinement and surface current) than the traditional LC resonance of an SRR, further research needs to be directed at their response under various angles and polarizations of incident light to apply to the 3D inclinometer. Improvement of the 3D SRR structures is a topic of continued research within our group.

In conclusion, split-ring resonators (SRRs) illuminated by a THz wave at their resonant frequencies provide the means to realize small, reliable, low power angular sensors for applications in medical microbots. Attempts made to leverage the SRRs for navigation applications have been primarily hindered by the 180° isotropy of its response. The two-dimensional nature of the microscale fabrication techniques does not allow for overcoming this isotropy issue. Using the self-folding technique we have developed a three-dimensional cubic inclinometer with tilted split-ring resonator arrays that is capable of resolving rotations between 0° to 360° about all three axes. Resonators of three different lengths are chosen to distinguish between rotations, with an added tilt to break the 180° symmetry of the SRR response. For each rotation of cubic inclinometer about the X, Y or Z axis, there was found to be a unique combination of the transmission amplitude of L_1_, L_2_, and L_3_ resonators. The use of polymers for the fabrication of the panels and hinges, which act as the substrate for the Au SRRs, further ensures minimum disruption in the transmitted signal. Measurement of the transmission response at the resonant frequency of each of the resonators allows resolving the angle of rotation. Although, the fully anisotropic 3D structure does not contain a wide linear range, however, it introduces a new concept and provides an innovative technique for remotely determining the angular motion of a microbot while overcoming the limited range of current optical inclinometers. Additionally, the THz resonators require only a 20 ps pulse, with our current experimental setup if an automated measurement system was added measurements down to 1.6 °/ns can be possible. Before the commercialization of these devices can be achieved further investigation into the modification of the design of these devices may be necessary to improve the quality factor and achieve a larger change in transmission for smaller angles of rotation. Further research into the design of the introduced 3D anisotropic structures and terahertz detectors has the potential for the development of a new class of THz devices that leverage the anisotropic response of these metamaterials including triaxial optical inclinometers that can sense rotation changes down to 1°/ns.

## Methods

### Terahertz time-domain spectroscopy

A commercial GaAs emitter (Tera-SED, Laser Quantum) illuminated by a Ti:sapphire laser pulse train with 780 nm center wavelength, 80 MHz repetition rate, and 90 fs pulse width (MaiTai XF, Newport Corporation) was used to measure the optical response of the cube. Terahertz (THz) time-domain spectroscopy (TDS) was performed between 0.26–0.8 THz with a single-cycle ps pulse. The cube was placed on a precision rotation mount (RSP1, ThorLabs) of black anodized aluminum with a circular aperture of diameter 3.8 mm, by means of a Scotch double sided tape that is transparent to THz radiation. The mount provided the ability to rotate from 0° to 360° in 2° increments. The p-polarized light was allowed to enter the aperture and illuminate the cube, following which it was detected with an electro-optic sampling method using a 1 mm thick zinc telluride (ZnTe) crystal (INGCRYS Laser System Ltd.). The transmission was first measured for a cube without any resonators followed by a measurement of the cubes with the Au SRRs patterned on the surface; the two results were divided by each other to normalize the transmission response, thus further eliminating any substrate interference effects. The 11 mm^2^ aperture of the mount generates a cut-off frequency around 0.07 THz, generating a larger noise below that frequency. Since, all the resonators have a fundamental resonant frequency greater than 0.3 THz, this was not considered to be a problem. Minor differences in the position of the reference cube and the cube with SRRs cause the transmission response to be >1.0 at frequencies below 0.3 THz in Fig. 6b,c. A 10% error may be expected due to position mismatch between the reference un-patterned cube and the cubic sensor caused by manually placing the cubes on the aperture.

### Finite element modeling of the transmission response

The simulation of the transmission response of the 2D SRR on a 48 µm substrate and the cubic sensors of length 110 µm was performed using High Frequency Structural Simulator, HFSS, (version 13.0.1, ANSYS). The software uses an FEM technique where the 3D structure is divided into tetrahedral elements that are refined over several recursive calculations to produce a fine mesh. Solutions to the Maxwell’s equation are found producing an S-matrix where the S_21_ parameter provides the transmission characteristics. The 2D Au resonator with the specified length (36 µm), width (4 µm), thickness (300 nm) and gap (4 µm) was created on a Si substrate (48 × 48 × 10 µm^3^). The structure was then encapsulated by a vacuum box (96 × 96 × 96 µm^3^), and the excitation ports were applied to the top and bottom of the cube. Resonators of varying lengths were first simulated individually; the length was chosen such that even when varying the polarization directions for the incident wave (changing the tilt angle from 0° to 360°), the fundamental resonant frequency of no two resonators approached the same point (Supplementary Fig. S1). The cubic sensor had Au resonators of varying length (fixed gap, width, and thickness) designed on a hollow cube (110 × 110 × 110 µm^3^) of thickness 10 µm, and encapsulated within a vacuum box (200 × 200 × 200 µm^3^) with the same polarizations as the 2D structure.

Intrinsic material properties available in the software were used for Au, Si, and vacuum. Rotations about each axis were performed in steps of 15°. A frequency sweep from 0.02–2.0 THz in steps of 0.01 THz was run for a mesh refined over 20 adaptive passes with an error tolerance of 0.02 for the S-parameter. The resulting S_21_ parameter in decibels (dB) was plotted against the frequency to determine the transmission behavior of the structures. The animation of the surface current was used to evaluate the mode of each resonator. A 10% error in the simulation response for the rotations is also taken into account due to difference in position within the finite vacuum box on rotating the structure.

The simulation of the fabricated cube (500 × 500 × 500 µm^3^) of SU-8 thickness 20 µm with a 5 × 5 array of 72 µm resonators, a 6 × 6 array of 54 µm resonators, and a 9 × 9 array of 36 µm resonators, all Au, was performed using Ansys Electromagnetics Suite 16.0.0 with a distributive solve over an MPI cluster. Simulation for the fabricated structure was performed under port conditions for a frequency range from 0.03 THz to 2.0 THz in steps of 0.02 THz. The electric field (E) was oriented along the Y-axis and the magnetic field (H) was oriented along the X-axis, having placed the structure inside a vacuum box. The boundary and port conditions were applied to a vacuum box (600 × 600 × 600 µm^3^) keeping all the other parameters same. The SU-8 permanent photoresist was modeled using the commercial parameters provided by MicroChem Corp with relative permittivity = 4.1, dielectric loss tangent = 0.015, mass density = 1187 kg/m^3^, and resistivity 2.8 × 10^16^ Ωcm. The electrical conductivity of Au was taken to be 4.1 × 10^7^ S/m. The SPR 220 hinges were ignored during the simulation due to computational limitations and the large distance between the resonators and the hinges which minimizes the impact of the polymeric hinge material on the transmission response. When the 500 µm cube is rotated, the time and memory required for simulating the structure significantly increases. Hence, only the 0° position of the cube (Supplementary Fig. S4) was simulated.

### Fabrication process for a micro-scale 3D (cubic) sensor

A 10 nm Chromium (Cr) adhesion layer followed by 100 nm Copper (Cu) sacrificial layer were deposited on a commercial silicon wafer using Electron-Beam (E-Beam) evaporation (Supplementary Fig. S5a). Then SRR array patterns were transferred from a glass-mounted mask (designed in Autodesk AutoCAD) to Cu sacrificial layer with S1813 positive photoresist (MICROPOSIT) using photolithography process. This was achieved by spin-coating S1813 at 2000 rpm (for thickness of 1.8 μm), followed by a soft-bake at 115 °C for 1 min. S1813 was then UV-exposed in a mask aligner and developed using MF-319 developer (MICROPOSIT) for 90 s with agitation. 300 nm Gold (Au) SRRs were deposited by filling S1813 pattern using Au electroplating process with Tecnhi Gold 25 ES solution (Technic) (Supplementary Fig. S5b). After removing S1813 with acetone, SU-8 panels were patterned on top of Au SRRs by spinning SU-8 2010 (MicroChem) at 4000 rpm, which provides a 10 μm-thick SU-8 layer, followed by a soft-baking of 95 °C for 2 min 30 s. The sample was then exposed in a mask aligner followed by a post-baking of 95 °C for 3 min 30 s. SU-8 panels were developed in SU-8 developer (MicroChem) for 2 min 30 sec and then transferred to a hot plate and hard-bake at 200 °C for 15 min to further cure the photoresist and anneal the cracks on the surface of the photoresist. After hard bake, the mechanical and chemical properties of SU-8 panels can be preserved and can survive high temperature exposure during the self-folding process (Supplementary Fig. S5c). The hinge of the cube is made of SPR 200–7.0 positive photoresist (MEGAPOSIT). SPR 220 was first spun on the sample at 1000 rpm followed by another spin coating of SPR 220 with the same rpm, which results in a thickness of 21 μm. After the sample was left undisturbed for 3 min to even the photoresist on top of the sample, the following baking process was conducted: 60 °C for 30 s, 115 °C for 90 s and 60 °C for 30 s. SPR 220 was left undisturbed for 3 h and then exposed in a mask aligner followed by developing in AZ developer for 110 s (Supplementary Fig. S5d). The 2D structure was thus defined with 6 SU-8 panels, Au SRR arrays on each face of the panel and SPR 220 hinges between and around panels (Supplementary Fig. S5d). The sample was then immersed in APS Copper etchant 100 (Transene) to release the 2D structure from the silicon wafer by etching the Cu sacrificial layer (Supplementary Fig. S5e). The 2D structure was then transferred from Cu etchant to DI water for the self-folding process. During the self-folding process, water with 2D structures was placed on a hot plate. Hot plate temperature was gradually increased (every 10 °C) from 100 °C to 300 °C until water is boiled. The SPR 220 hinge reflows under high temperature and generates stress between panels to fold the structure (Supplementary Fig. S5f). Upon cooling, SPR 220 hinge became solid again and secure the 3D cubic structure (Supplementary Fig. S5f). The self-assembly of the cubic structure can be seen in the video file available with the Supplementary Information, which shows the folding of the 2D into the 3D cube when partially released from the substrate. The self-assembly can occur in either air or water. In order to maximize transmission and to minimize the noise, the cube fabricated had to have a length that was close to the aperture of the light source to be used for measurement. Using the self-folding technique, we were able to fabricate cubic sensors of length, 500 µm (Fig. 5). Due to the decay in transmission of these resonators when tilted about their axis, the tilt was chosen so as to obtain a high transmission at the initial position of 0°. The resonators measuring 72 µm, 54 µm, and 36 µm are titled at angles 20°, 15°, and 10° about their axis, respectively.

## Electronic supplementary material

Supplementary Information

Video of Self-Assembly
