# Supplementary material for: Three-Dimensional Anisotropic Metamaterials as Triaxial Optical Inclinometers
Source: Sci Rep. 2017 Jun 2;7:2680. doi: 10.1038/s41598-017-02865-z (PMC5457403; doi:10.1038/s41598-017-02865-z)
Supplement: Supplementary file 1 — Supplementary Information [file 41598_2017_2865_MOESM1_ESM.pdf]

Supplementary Information

Three-Dimensional Anisotropic Metamaterials as Triaxial Optical Inclinometers

Kriti Agarwal, Chao Liu, Daeha Joung, Hyeong-Ryeol Park, Sang-Hyun Oh, and Jeong-Hyun Cho

Department of Electrical and Computer Engineering, University of Minnesota, Minneapolis, MN 55455, USA

E-mail: jcho@umn.edu

List of Contents

- Figure S1. Simulated frequency response of the individual 72 µm, 54 µm, and 36 µm resonators.......... 2
- Figure S2. Illustration of the cube under different rotation conditions.......... 3
- Figure S3. Simulated response of the 3D inclinometer for the 180° rotations…............... 4
- Figure S4. Simulation results for the cube with lengths matching the fabricated cube…. 5
- Figure S5. Illustration and optical images of self-folding cubic SRR fabrication process.......... 6
- Self-assembly video description.......... 7

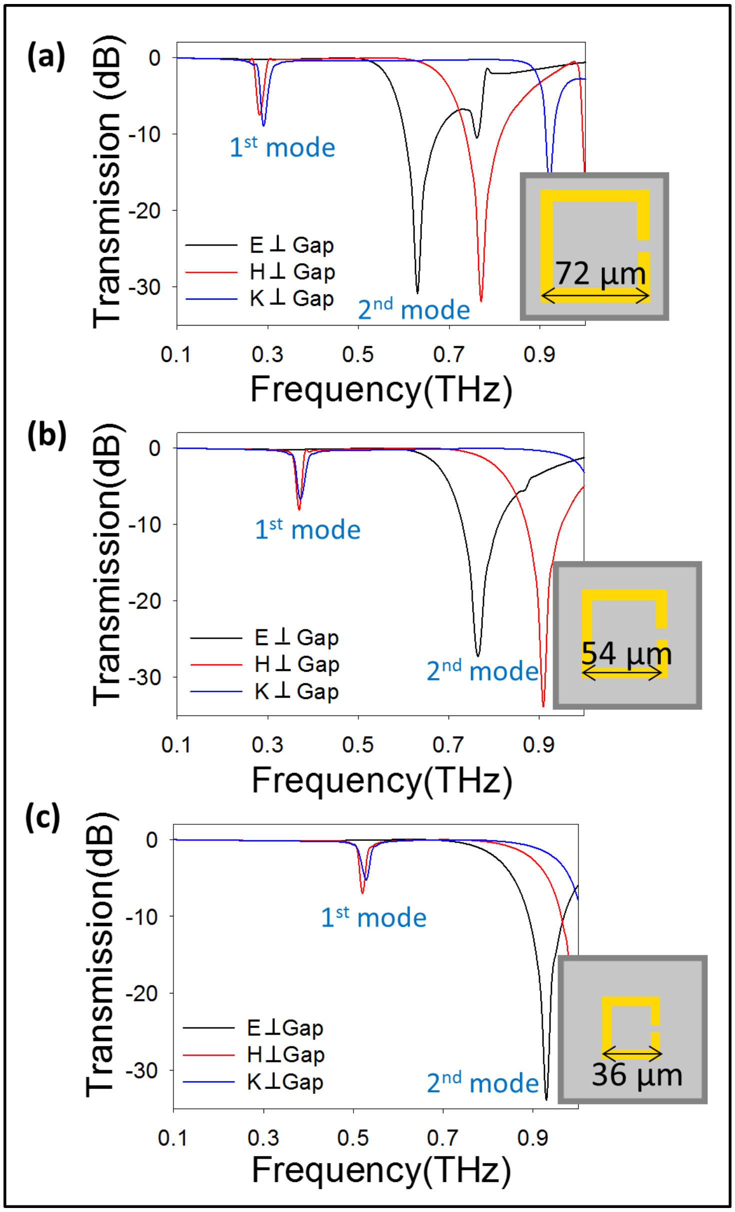

Figure S1. **Simulated frequency response of the individual 72 µm, 54 µm, and 36 µm resonators.** Transmission curves displaying the resonant frequency of the various modes induced within the resonators when the magnetic field (H) is perpendicular to the gap (1st Mode), electric field (E) is perpendicular to the gap (2nd Mode), and the wave vector (k) is perpendicular to the gap (plane of magnetic field perpendicular to SRR) for resonators of length (a) 72 µm, (b) 54 µm, and (c) 36 µm.

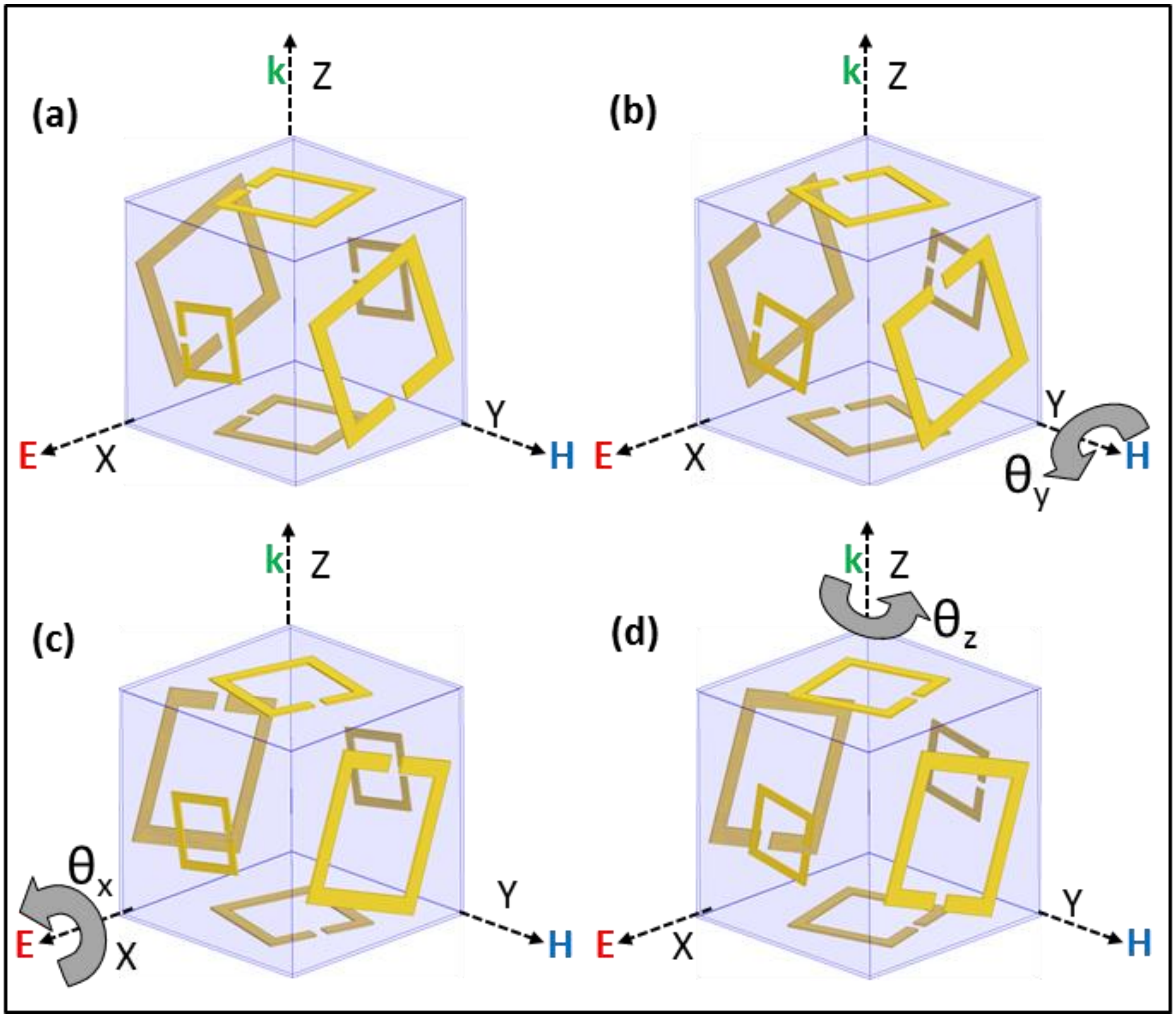

Figure S2. Illustration of the cube under different rotation conditions. Illustration of the cubic sensor and the orientations of each resonator when the cube is at, (a) Initial Position ($\theta_x = \theta_y = \theta_z = 0^{\circ}$) (b) Rotation of 180° about Y-axis ($\theta_y = 180^{\circ}$) such that L_1 is now rotated at 180° compared to initial position (c) Rotation of 180° about X-axis ($\theta_x = 180^{o}$) such that L_1 is now rotated at 140° compared to initial position. (d) Rotation of 180° about Z-axis ($\theta_z = 180^{o}$) such that L_1 is now rotated at 320° compared to initial position.

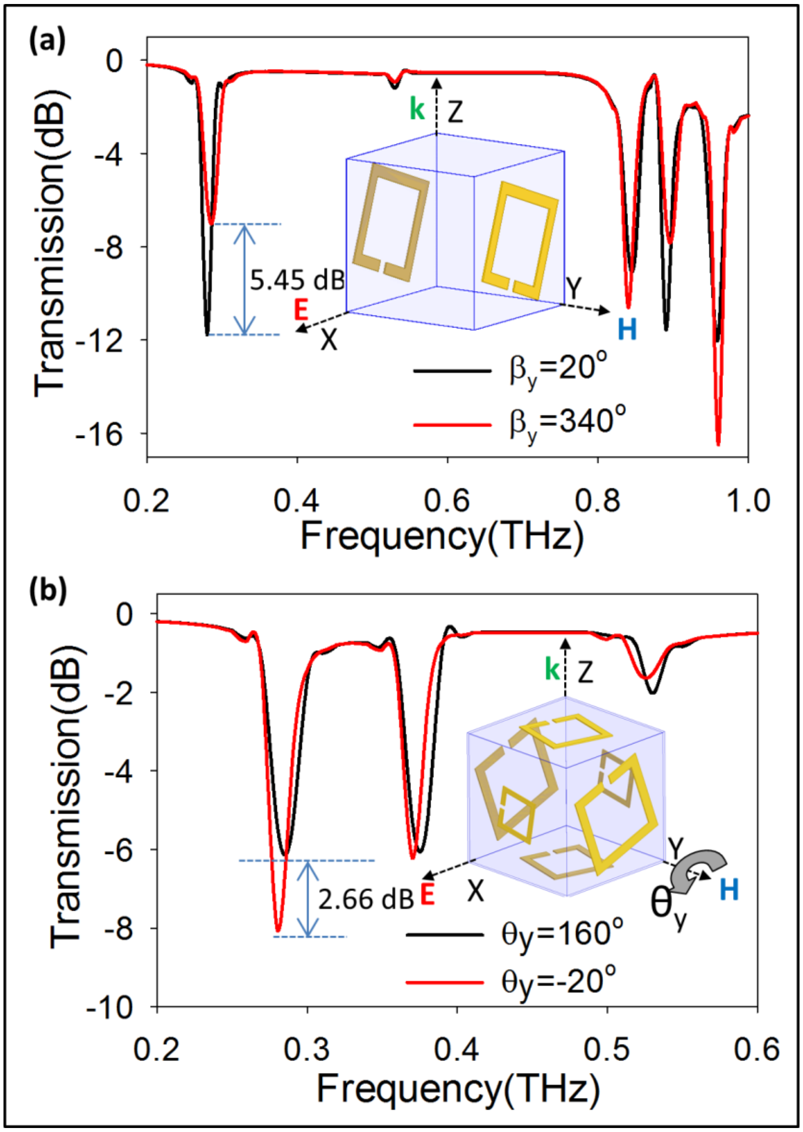

Figure S3. Simulated response of the 3D inclinometer for the 180° rotations. (a) Simulated transmission response of the cube with only L_1 resonator of length 72 μm placed on it, showing the variation in resonance between 0° and 340° when polarized with the wave vector parallel to the plane of the resonator, and the magnetic field polarized (H) perpendicular to the plane of the resonator. (b) Simulated transmission response of the cubic structure with varying resonator length shown in Fig 1c, demonstrating the large transmission amplitude change at resonance of 2.66dB L_1 resonator, at a rotation of 160°, and -20° about the Y-axis when the L_1 resonator enters the original βy=0° and 180° configurations.

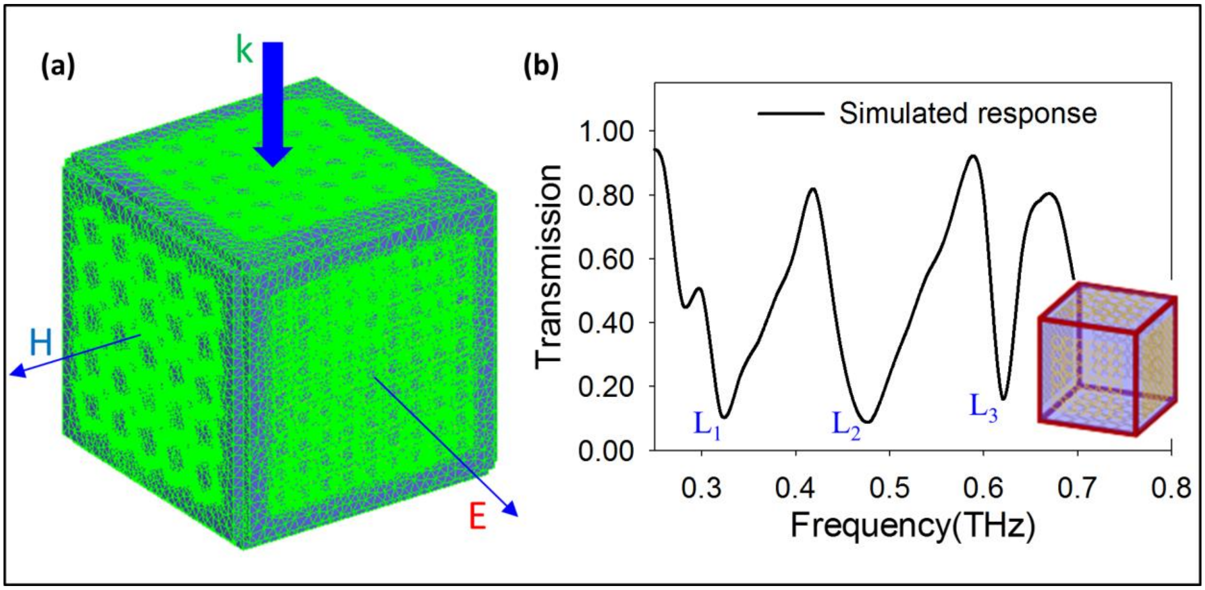

Figure S4. Simulation results for the cube with lengths matching the fabricated cube. (a) Simulated cube showing the mesh, and polarization direction used for simulation of the transmission spectrum. (b) Simulated transmission response of the cubic sensor at 0° (initial position) with length, a=500 µm, and tilted resonator arrays of length 72 µm, 54 µm and 36 µm patterned on its surface.

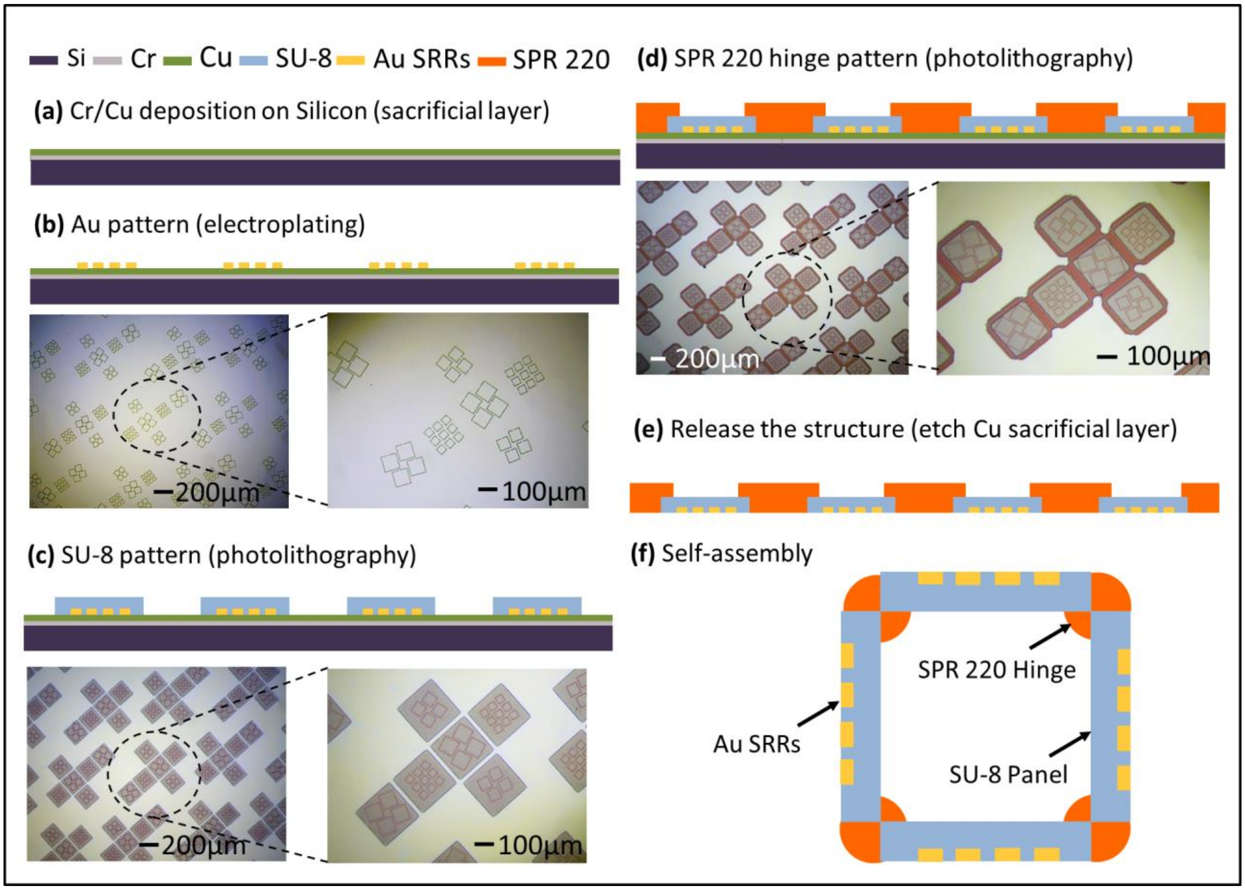

Figure S5. Illustration and optical images of self-folding cubic SRR fabrication process. (a) Deposition of Cr/Cu sacrificial layer by evaporation on a Si substrate. (b) Electroplating of the Au SRR and patterning by lift-off process. (c) SU-8 deposition and patterning by photolithography for the panel. (d) SPR 220 deposition and patterning by photolithography as the hinge material. (e) Etching of the sacrificial layer to release the 2D structure from the substrate. (f) Self-assembly of the released 2D structure to form a 3D cube by heating to about 100 °C.

Self-Assembly Video

The supplementary AVI video file was captured during the self-folding process of the 500 μm sized cubes with SU-8 panels and SPR 220 hinges. The video was captured during self-folding in air, however, the same process can be observed in water as well.
